# Regulatory network analysis reveals the oncogenesis roles of feed-forward loops and therapeutic target in T-cell acute lymphoblastic leukemia

**DOI:** 10.1186/s12920-018-0469-0

**Published:** 2019-01-15

**Authors:** Mengxuan Xia, Qiong Zhang, Mei Luo, Pan Li, Yingxue Wang, Qian Lei, An-Yuan Guo

**Affiliations:** 1 Hubei Bioinformatics and Molecular Imaging Key Laboratory, Department of Bioinformatics and Systems Biology, Key Laboratory of Molecular Biophysics of the Ministry of Education, College of Life Science and Technology, Huazhong University of Science and Technology, 1037 Luoyu Road, Wuhan, 430074 China; 2 Department of Hematology, the Second Hospital of Shandong University, Jinan, 250033 Shandong China

**Keywords:** T-ALL, Pathogenesis, Cell cycle, Cell proliferation, Feed-forward loops

## Abstract

**Background:**

T-cell acute lymphoblastic leukemia (T-ALL) is an aggressive hematological malignancy. Aberrant expressed genes contribute to the development and progression of T-ALL. However, the regulation underlying their aberrant expression remains elusive. Dysregulated expression of transcription factors and miRNAs played important regulatory roles in the pathogenesis of T-ALL.

**Methods:**

In this study, we analyzed the alteration of transcriptome profiling and regulatory networks between T-ALL sample and normal T cell samples at transcriptional and post-transcriptional levels.

**Results:**

Our results demonstrated that genes related to cell cycle and cell proliferation processes were significantly upregulated in T-ALL comparing to normal samples. Meanwhile, regulatory network analyses revealed that *FOXM1*, *MYB*, *SOX4* and miR-21/19b as core regulators played vital roles in the development of T-ALL. *FOXM1*-miR-21-5p-*CDC25A* and *MYB/SOX4*-miR-19b-3p-*RBBP8* were identified as important feed-forward loops involved in the oncogenesis of T-ALL. Drug-specific analyses showed that GSK-J4 may be an effective drug, and *CDC25A*/*CAPN2*/*MCM2* could serve as potential therapeutic targets for T-ALL.

**Conclusions:**

This study may provide novel insights for the regulatory mechanisms underlying the development of T-ALL and potential therapeutic targets.

## Background

T-cell acute lymphoblastic leukemia (T-ALL) is an aggressive hematological malignancy [1], which accounts for 10–15% of pediatric and 25% of adult ALL cases [2]. The anomalous genetic and epigenetic reprogram could arise abnormal proliferation of T lymphoid blasts and transform T-cell precursors into malignant T-ALL lymphoblasts [3]. However, the detail regulatory mechanisms underlying the transformation procedure are still unknown. Thus, a better understanding of the biological regulation involved in the T-ALL pathogenesis is necessary for identifying strategies of prevention and therapy.

Previous studies reported that dysregulation of transcription factors (TFs) to target genes could block myeloid and hematopoietic stem cells differentiation, eventually causing acute leukemia. *HOXA9* as an important TF plays vital roles in hematopoietic stem cell expansion and is commonly dysregulated in human acute leukemia [4]. Besides, *HOXA9* could co-regulate with *CEBPA* to suppress the expression of *CDKN2A* and *CDKN2B* to increase cell proliferation [5]. *MYC* is a critical oncogenic TF in T-ALL, the overexpression of *MYC* and *CDKs* maintain the leukemic growth by promoting cell proliferation and initiating DNA replication [6]. *CHK1* promotes proliferation and viability of T-ALL by down-modulating replication stress and preventing *ATM*/caspase-3-dependent cell death [7]. Additionally, overexpression of *USP44* contributes to the pathogenesis of T-ALL by regulating *CDC20*-*APC/C* activity and chromosome instability [8].

MicroRNAs (miRNAs) are a category of small noncoding RNAs play important roles in T-ALL [9]. For instance, the downregulation of miR-101 promotes the expression of *TAL1*, whose high expression induces stem-cell-like transcriptional circuitry in T-ALL [10]. Overexpression of miR-1246/1248 could regulate NOTCH2 pathway to promote cell proliferation in the T-ALL [11]. Moreover, TFs and miRNAs may co-regulate target genes to form feed-forward loops (FFLs), which serve as critical motifs in gene regulatory networks and play critical roles in multiple biological processes [12, 13]. The FFLs could reduce the complexity of regulatory networks and provide comprehensive clues to identify oncogenes and the underlying regulatory mechanisms [14]. *BRCA1* and miR-25 targeting *ADAMTSL3* led to better survival in colorectal cancer [15]. TF *STAT5A* and miR-146b-5p co-regulated the expression of *NUMB* to play important regulation on leukemogenesis by enhancing the ROS level and genome instability [16]. miR-19b represses *CYLD* expression and upregulates *NFKB* expression to active NF-KB pathway in T-ALL, and *TCF3* regulates miR-125b and *MYC* in AML pathways [17, 18]. Thus, dissecting regulatory networks and exploring FFLs consisted of TF-miRNA-targets could provide profound insights to reveal the molecular pathogenesis of T-ALL.

In this study, we analyzed the alteration of transcriptional profiling including genes and miRNAs between T-ALL and normal T cells. Functional enrichment and TF-miRNA regulatory network analyses identified that *FOXM1*, *MYB*, *SOX4* and miR-21/19b as core regulators to regulate the cell cycle related processes. Besides, *CDC25A* and *CAPN2* may be potential targets for the treatment of T-ALL. This work will be helpful to enhance the understanding of pathogenesis as well as therapy for T-ALL.

## Methods

### Data sources and differential expression of miRNAs and genes

For the mRNA gene expression, we selected the GSE48558 dataset from GEO database (15 T-ALL cell lines, 13 T-ALL patient samples and 17 normal T cell samples), in which T-ALL cell lines included CEM, JURKAT, MOLT and KARPAS45 [19]. GEO2R [20] was used to compare the gene expressions of T-ALL cell lines and T-ALL patient samples with normal T cells, respectively. Benjamini & Hochberg method was used to adjust the *p*-value, and *p*-value <1e-5 and fold change (FC) > 4 were considered as significant difference. The intersection of these two comparisons was considered as differentially expressed genes (DEGs) of T-ALL and normal samples. Then, the DAVID Tool [21] was applied to perform enrichment analysis for upregulated and downregulated DEGs, respectively.

miRNA datasets were obtained from GSE89978 (48 T-ALL patient samples, 2 CD4^+^ CD8^+^ and 2 CD34^+^ healthy donor samples) [22]. In these datasets, T-ALL lymphoblasts collected from the blood samples and bone marrow of T-ALL patients, and CD4^+^ CD8^+^/CD34^+^ cells were obtained from healthy donor thymocytes. Using DESeq2, we compared the T-ALL samples with CD4^+^ CD8^+^ and CD34^+^ normal samples, respectively. We required FC > 2, *p*-value < 0.05 and read count > 100 in at least 1 sample as the cutoffs for differential expression. To avoid the bias caused by the unbalanced sample size in the different groups, RNentropy was employed to evaluate the robust of miRNAs expression, and the miRNAs with convergent expression profiles were used for further analyses [23]. Finally, we selected miRNAs overlapped in two comparisons as differentially expressed miRNAs (DEMs). The MeV (https://sourceforge.net/projects/mev-tm4/files/) software with hierarchical clustering method was used to present the expression heatmap by showing the mean expression.

### Generation of network and analysis of hub components

Briefly, the miRNA-TF-target regulatory network was constructed by the following three components: 1) TF-targets regulation. TF-targets regulatory information was obtained from the ChIP-Seq experimental datasets of public databases (ENCODE, and hTFtarget http://bioinfo.life.hust.edu.cn/hTFtarget/) and predictive TF-targets from AnimalTFDB [24] and UCSC; 2) miRNA-targets regulation. Experimentally validated miRNA-targets regulations were incubated from miRTarBase and TarBasev7.0, while the predictive regulations were collected form Targetscan and miRanda; 3) Regulatory network detection. The detailed procedures about how to select nodes to form potential FFLs were described in our previous studies [14, 25]. In this study, the DEMs and DEGs were used to build the regulatory network, and all networks were visualized by Cytoscape (version 3.4.0).

NetworkAnalyzer was employed to calculate the degree of nodes in the network and helped to identify the hubs. The subnetwork was built by the DEGs enriched in the GO terms and the corresponding regulatory DEMs and TFs. Based on the subnetwork, the top 5 TFs and miRNAs that contained the maximum connection combined with their targets were extracted to find the core TFs and miRNAs. The half maximal inhibitory concentration (IC50) information of 746 drugs and the corresponding genes expression profiles of 1861 cell lines were collected from GDSC [26] and CTRP [27] databases. The spearman correlation coefficient (cor_sprm) between the IC50 and genes expression profiles were calculated in all cell lines using the GSCALite [28], and the drug-gene pair with the *p*-value <1e-4 & |cor_sprm| > 0.4 was considered as significant one for the further analysis.

## Results

### Transcriptional profiling analysis reveals significant upregulation of cell proliferation and downregulation of immune response in T-ALL

**Fig. 1:**
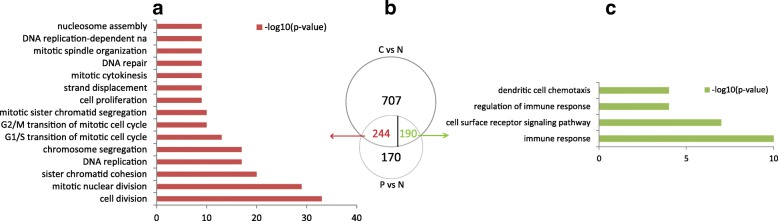
Differential expression analysis in T-ALL vs normal T-cells. (**a**) GO enrichment results of upregulated DEGs (red). (**b**) Venn graph of DEGs in the two comparisons. C: T-ALL Cell lines, N: Normal human T-cells, P: T-ALL patients. Numbers in the sectors are the numbers of DEGs downregulated (green) and upregulated (red). (**c**) GO enrichment results of downregulated DEGs (green)

**Fig. 2:**
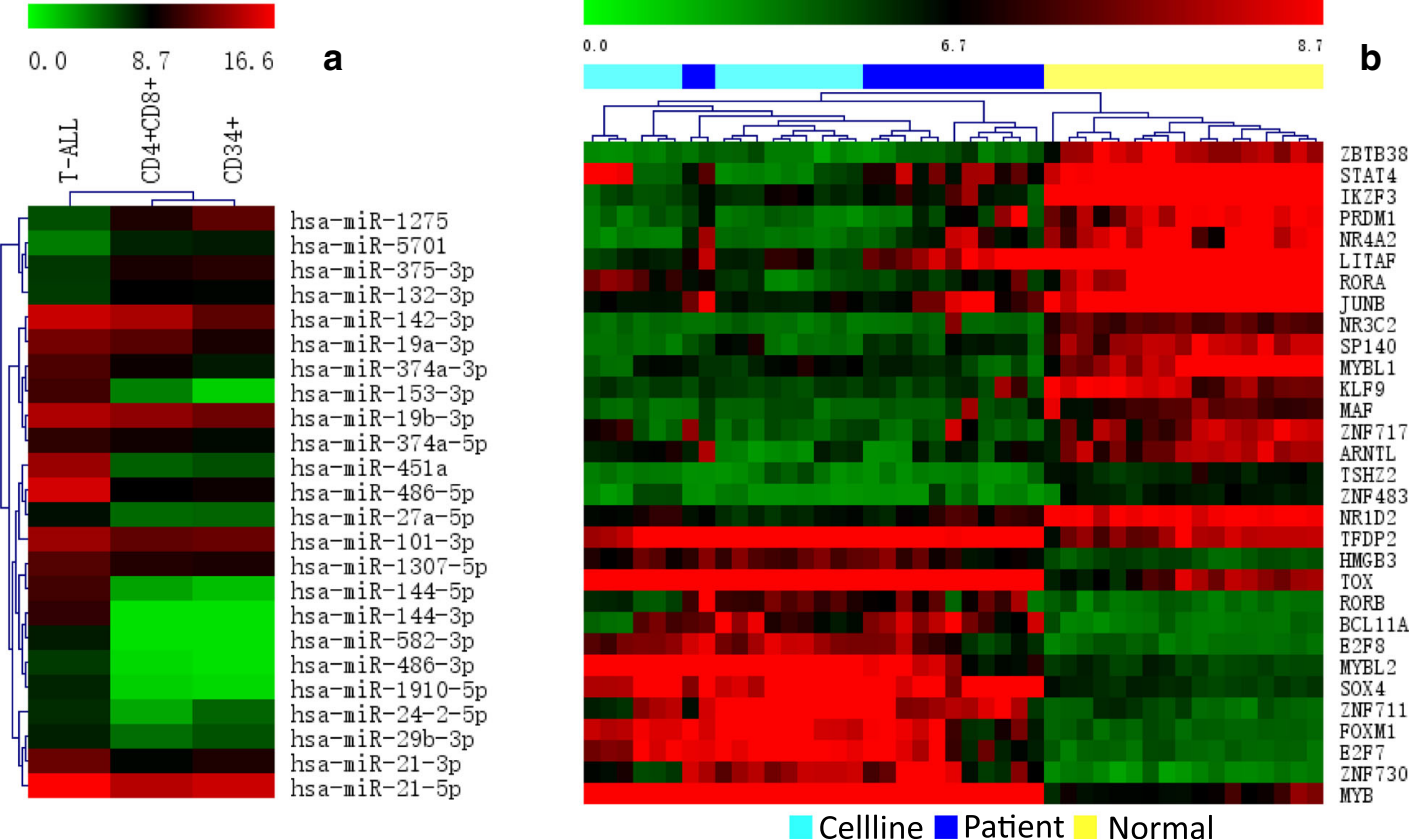
(**a**) Hierarchical clustering of miRNAs significantly differentially expressed in the comparisons of T-ALL samples (patients and cell lines) vs normal T cells (**b**) Hierarchical clustering of TFs. Upregulation in red and downregulation in green

To explore the alterations of transcriptional profiling involved in the progress of T-ALL, we detected the DEGs and DEMs by comparing T-ALL samples with normal samples. As a result, we identified 1141 and 604 DEGs in the comparisons of T-ALL cell lines vs normal T-cells and T-ALL patients vs normal T cells, respectively. There were 434 DEGs overlapped with the same trends (244 upregulated and 190 downregulated) in the two comparisons (Fig. 1b). Additionally, we found 24 DEMs in the comparison of T-ALL samples vs normal T cells (Fig. 2a). Among them, 20 miRNAs were upregulated and 4 miRNAs were downregulated in T-ALL.

For further investigating the functions of DEGs in T-ALL, we performed functional enrichment analysis on the DEGs. Interesting, more than 50% (130/244) upregulated DEGs were enriched in the processes related to cell cycle, such as cell division, cell proliferation and DNA replication (Fig. 1a). Uncontrolled cell cycle and cell proliferation led to the abnormal cell growth of T-cells [29], which suggested that these genes played important roles in the development of T-ALL. The downregulated genes were enriched in immune related processes and cell surface receptor signaling (Fig. 1c), which implied that the immune system of T-ALL patients was suppressed.

### Regulatory network involved in the development of T-ALL

TFs are key regulators of gene expression and play vital roles in the pathogenesis of T-ALL. Among the 434 DEGs, 31 differentially expressed TFs (13 up and 18 down) were identified and their expression hierarchical clustering analysis was showed in Fig. 2b. For example, the downregulation of *MYB* and *SOX4* could suppress the migration, cell proliferation and development of T-ALL [30, 31]. Meanwhile, downregulation of *JUNB* by miR-149 promoted T cell proliferation and suppressed apoptosis [32].

**Fig. 3:**
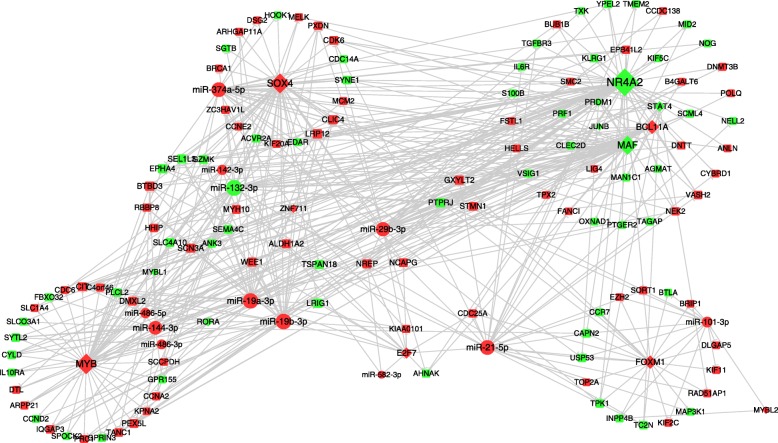
The regulatory network of DEGs and DEMs. Green, downregulated genes and miRNAs. Red, upregulated genes and miRNAs. The diamond nodes, TFs; Ellipse nodes, DEMs; Round Rectangle, DEGs. The size of the nodes represents the degree of the nodes

For understanding the regulation relationships among TFs, miRNAs and their targets, we constructed the regulatory network based on the DEGs and DEMs. Our network contained 486 edges that consisted of 132 DEGs (14 TFs and 118 genes) and 12 miRNAs (Fig. 3). Meanwhile, *NR4A2*, *MYB*, *SOX4* were the top 3 TFs in the connected degree, and *FOXM1* was the only TF enriched in cell cycle related pathways, while *FOXM1*, *MYB* and *SOX4* regulated about the amount of 66% of the genes and all the miRNAs in the network. miR-21-5p, miR-19b-3p and miR-132-3p were the top 3 miRNAs which regulated about 66% of the genes in our network. In conjunction with the results above, the hub TFs and miRNAs combined with their target genes in our regulatory network may form key modules involved in the development of T-ALL.

### Key regulatory modules participate in the progression of T-ALL

**Fig. 4:**
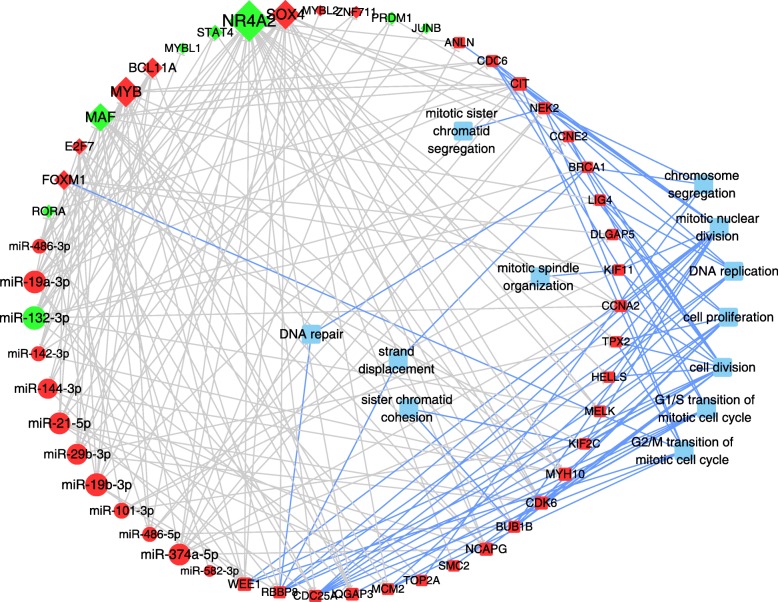
TF and miRNA regulatory subnetwork of cell cycle and cell proliferation genes. Light blue Round Rectangle, enriched GO terms; Grey line, regulatory relationship of TFs and miRNAs to genes; Blue line, relationship of mRNAs to GO terms. The means of nodes are the same as Fig. 3

To reveal how the hub regulators affect cell proliferation and result in T-ALL, we reconstructed a subnetwork using hub genes which enriched in cell cycle/proliferation processes and were with high connection property to TFs and miRNAs (Fig. 4). Totally, 51 nodes with 69 FFLs were contained in the subnetwork. We detected 3 hub TFs (*FOXM1*, *MYB* and *SOX4*), which cooperated with 11 miRNAs to regulate most genes (18/25) in these subnetwork.

**Fig. 5:**
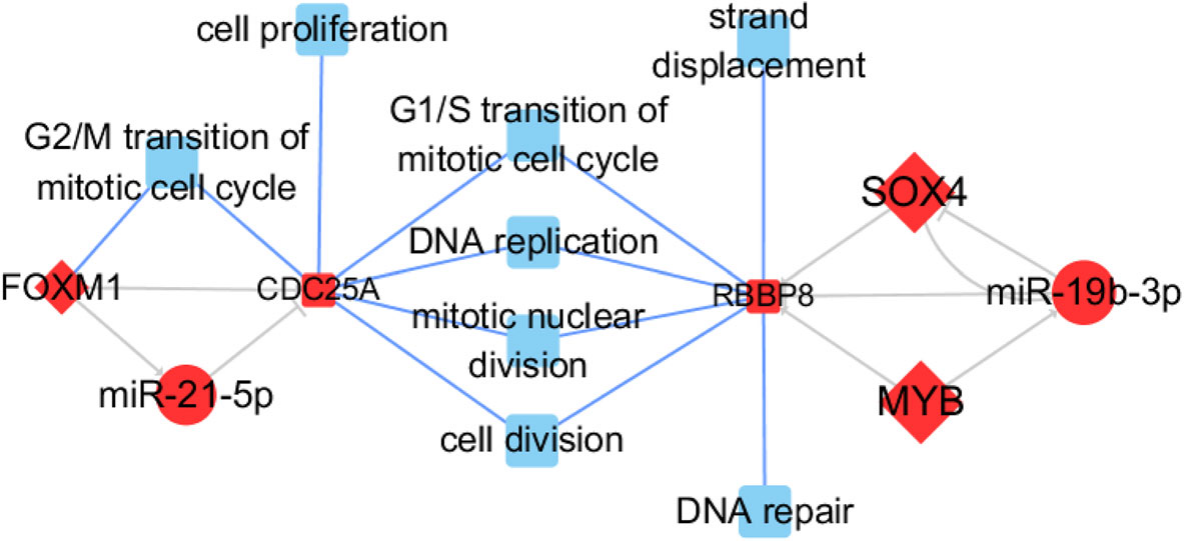
FFLs for *FOXM1*-miR-21-*CDC25A* and *MYB*/*SOX4*-miR-19b-*RBBP8* and their enriched GO terms. The means of nodes are the same as Fig. 3

To further explore the functions of key regulators, we dissected the subnetworks and extracted important FFLs which may play vital roles underlying the development of T-ALL (Fig. 5). The FFLs *FOXM1*-miR-21-5p-*CDC25A* and *SOX4*-miR-19b-3p-*RBBP8* were predicted to regulate cell cycle and cell division. *FOXM1* could regulate the expression of target gene *CDC25A*, which was considered as a symbol to characterize the accumulation of G1/S and early S phase cells and the downregulation of *CDC25A* induced cell cycle arresting in T-ALL [33]. Meanwhile, inhibition the expression of *FOXM1* could decrease the proliferation of Jurkat cells and improved the survival of children T-ALL [34]. *SOX4*/*MYB* could regulate the expression of *RBBP8*, which play important roles in DNA repair [35].

**Fig. 6:**
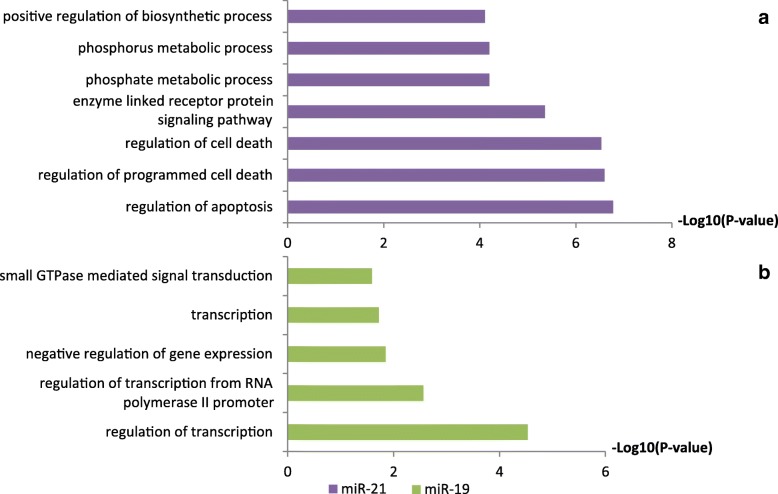
GO terms enrichments result for the target genes of miR-21-5p (**a**) and miR-19b-3p (**b**)

To investigate the possible functions of miR-21-5p and miR-19b-3p in the FFL and the development of T-ALL, we analyzed the target genes of the two miRNAs using DAVID. Interestingly, the functions of targets genes for miR-21-5p were focused on regulation of apoptosis and cell death processes (Fig. 6a), which demonstrated that miR-21-5p played important roles in cell cycle regulation. Interestingly, miR-21-5p was observed overexpressed in both early and late-stage primary murine T-ALL cells, while knocked down of miR-21-5p induced T-ALL cell apoptosis [36]. Furthermore, miR-21-5p could regulate *CDC25A* participating the cell cycle process [37]. Meanwhile, the targets of miR-19b were mainly related to regulation of transcription (Fig. 6b), and miR-19b could induce leukemogenesis under *NOTCH1* overexpression [38]. Thus, the above results illustrated, *FOXM1*-miR-21-5p-*CDC25A* and *SOX4*-miR-19b-3p-*RBBP8* may serve as core regulatory modules involved in the development of T-ALL.

### Potential drug indicators of genes in the regulatory network

**Fig. 7:**
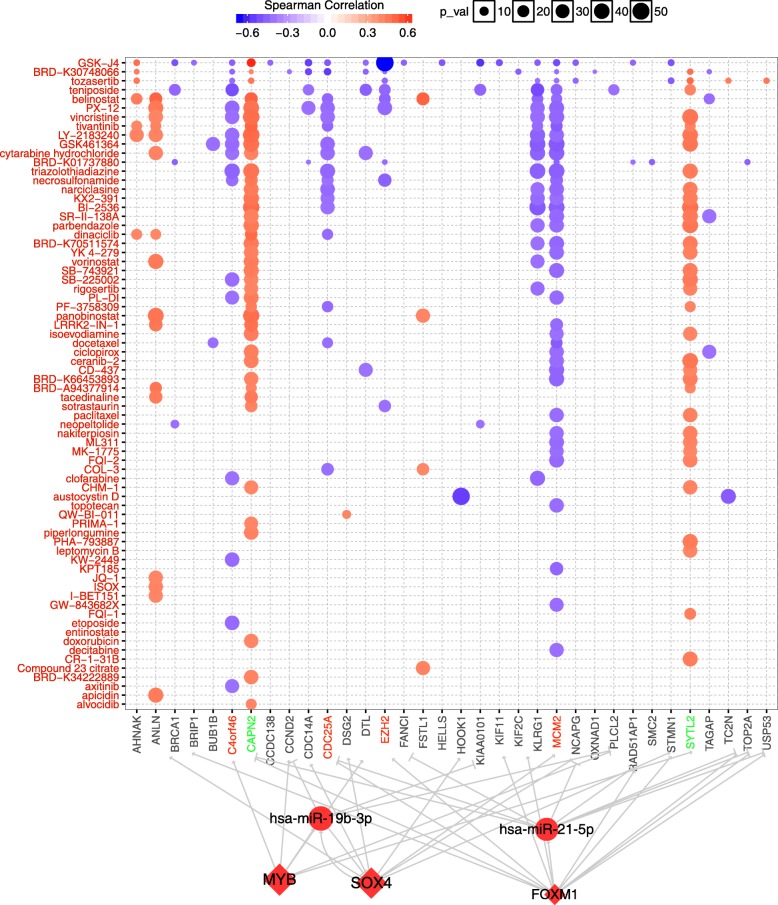
The correlation of drugs IC50 and genes in the regulatory network of *FOXM1*, *MYB*, *SOX4*, miR-21-5p and miR-19b-3p. Drug names are in red font. Upregulation significance genes are red; downregulation significance genes are green. Orange or purple dots mean positive or negative correlation between drugs IC50 and genes expression, respectively

For identifying the potential therapeutic indication of the regulatory network, we analyzed the correlation between IC50 of drugs and regulators in our network, and constructed the detail a drug-FFL regulatory network (Fig. 7). Totally, 34 genes were highly correlated with 72 drugs or small molecules, *ANLN*, *CAPN2, C4orf46*, *SYTL2*, *CDC25A*, *MCM2* and *KLRG1* were the most significant genes with highest correlation to more than 10 drugs. GSK-J4, BRD-K30748066, tozasertib and Teniposide were the top 4 drugs correlated with many genes. GSK-J4 as small molecule inhibitor could suppress the growth of T-ALL cells, and its target *KDM6A*/*UTX* acted as a pro-oncogenic cofactor in T-ALL [39]. Teniposide was reported in several drug combinations in treatment of T-ALL, showed ability to induce cell death [40]. These results implied that these genes may be efficient biomarkers and these drugs may be able to target these genes to treat T-ALL.

Besides, *CDC25A*, which identified as a core target gene of *FOXM1*-miR-21-5p-*CDC25A*, showed negative correlation with drug which inhibited T-ALL, such as GSK-J4 and Teniposide. Meanwhile, *CAPN2* was downregulated in T-ALL sample and positive correlated with 41 drugs. Beta carotene can increase the expression of *BAX* and *CAPN2* and lead to the apoptosis of AML cell lines [41]. Furthermore, abnormally high expression of *MCM2* was reported in recurrence of ALL [42], we found *MCM2* was upregulated in the T-ALL samples and showed negative correlation with 39 drugs, which implied *MCM2* may serve as a potential therapeutic target. Thus, our regulatory network may serve as a resource to explore the development of T-ALL and provide potential targets for drug treatment.

## Discussion

The T-ALL still remains a cancer with worse prognosis for high recurrence, understanding the regulatory mechanisms of T-ALL could provide comprehensive insights for the diagnosis and treatment. In current study, we investigated the potential regulatory mechanisms of the development of T-ALL through differential expression and network analysis across T-cells, T-ALL and normal samples. Our data demonstrated that the biological processes including cell cycle, DNA replication and cell proliferation were enhanced, but immune response was decreased in T-ALL. We found that *FOXM1*, *MYB*, *SOX4* and miR-21/19b as hub regulators may form FFLs with *CDC25A* and *RBBP8* to regulate cell cycle related pathways. Drug targets analysis could provide preliminary clues that *CAPN2*, *CDC25A* and *MCM2* may serve as potential therapeutic targets for the treatment of T-ALL.

Understanding the regulatory mechanisms underlying the development of T-ALL could provide profound insights to reveal the molecular pathogenesis of T-ALL. Most of the previous studies were focused on the effects of single gene or pathways in T-ALL, rare reports were involved in the regulatory relationships on systems level. Our results demonstrated that genes involved in the processes of cell proliferation, cell cycle and DNA replication were upregulated in the T-ALL samples (Fig. 1), which indicated the unexpected cell proliferation occurred in the T-ALL. Several researchers reported that cell proliferation, cell cycle and DNA replication pathways played crucial roles in T-ALL, and blocked down genes related to cell cycle could contribute to the remission of T-ALL. For example, resveratrol inhibited Akt/mTOR and activated p38-MAPK pathways could induce apoptosis of malignant cells in T-ALL [43]. Dysregulation for the genes involved in the DNA replication processes could cause differentiation blockade of normal T-cells, which in turn results in the initiation of T-ALL [44]. Our regulatory analysis showed that the TFs *FOXM1*, *MYB*, *SOX4* and miR-21/19b represented as hub nodes and core regulators in the T-ALL regulatory network (Fig. 4). In our key regulatory networks, the TF *FOXM1* and miR-21 predicted co-regulating target gene *CDC25A*, and formed a FFL participating the DNA replication, cell proliferation and division processes (Fig. 5), which may be a key module involved in the development of T-ALL. The TF *FOXM1* was reported as a core factor involved in the cellular transformation and tumor initiation by regulating the G1/S and G2/M transitions [45], while the miR-21 could modulate cell proliferation and migration [46]. Meanwhile, downregulation of *CDC25A* was concordant with the inhibition of Jurkat cell proliferation in a G2/M arrest [33]. Furthermore, the expression profiles of the *FOXM1*-miR-21-5p-*CDC25A* FFL loop was consistent with the above reports, which implied the vital regulatory roles of the FFL in the development of T-ALL. The upregulated expression of the TF *FOXM1* could increase the expression of both *CDC25A* [47] and miR-21-5p, whereas the miR-21 could target *CDC25A* [48], which in turn formed a dynamic balance for the FFL involved in the cell proliferation process, suggesting the miR-21 may mediate a negative feedback loop collaborated with the TF *FOXM1* to regulate their targets *CDC25A.* Consequently, although the miR-21 repressed the expression of *CDC25A,* the *CDC25A* maintained at a high expression level promoted by TF *FOXM1,* which may trigger the abnormal cell cycle and cell proliferation in the progress of T-ALL. Additionally, *SOX*-miR-19b-3p-*RBBP8* may reveal the regulatory relationships involved in the T-ALL as well. *SOX4* could enhance chondrogenic differentiation and proliferation of human synovium-derived stem cell [49], while downregulation of *SOX4* suppressed cell proliferation and induced apoptosis [50], which may contribute to remission of T-ALL. miR-19b was reported regulating cell cycle related pathways, and inhibited the activity of the PI3K-AKt signaling pathway leading to inactivation of P53 and cell growth in cancers [51]. *RBBP8*, also called *CtIP*, promoted DNA-end resection and maintained chromosome stability [35], and depletion of *CtIP* leading to cell arrested in G1 phase [52]. In conjunction of above results, *FOXM1*-miR-21-5p-*CDC25A* and *SOX*-miR-19b-3p-*RBBP8* FFL may regulate cell cycle related processes playing profound roles in the development of T-ALL.

Regulatory network combined with drug-specific analyses employed the gene expression, gene regulatory relationships and IC50 of drug to seek potential drug indicators involved in the development of T-ALL. Our data demonstrated that GSK-J4 showed high correlations with most genes in the regulatory network (Fig. 7), which implied that GSK-J4 may serve as a potential effective drug for T-ALL. Interesting, previous studies reported that GSK-J4 could decrease the activity of H3K27 demethylases *JMJD3*, which may inhibit *NOTCH1* related pathways and contribute to the anticarcinogen effects [53]. Meanwhile, gene *EZH2*, which was associated with H3K27 methylation and activated in dividing cells [54], showed highest negative correlation with GSK-J4. *EZH2* was highly expressed in several human tumors, whose upregulation promoted cell proliferation and amplified in cancer [55]. Additionally, increase the activity of *CAPN2* could eliminate malignant cells in ATL cells, and decrease of *CAPN2* expression was observed in ATL patients [56]. *FOXM1* and miR-21-5p co-regulated the expression of *CAPN2* in our FFL, which may be involved in the T-ALL. The expression level of *CAPN2* was downregulated in the T-ALL patients, and displayed high positive correlation with the drug GSK-J4, which suggested *CAPN2* may be acted as potential drug target in the T-ALL. Combined with above results, our drug indicators analysis may provide some potential drugs and targeted biomarker in the therapy of T-ALL.

## Conclusions

We analyzed the alteration of transcriptome profiling and regulatory networks between T-ALL sample and normal T cell samples at transcriptional and post-transcriptional levels. Our research identified some dysregulated genes enriched in cell cycle related processes. TF-miRNA co-regulatory network analysis demonstrated that *FOXM1*, *MYB*, *SOX4,* miR-21/19b and their FFLs *FOXM1*-miR-21-5p-*CDC25A* and *MYB/SOX4*-miR-19b-3p-*RBBP8* may play important roles in the cell proliferation of T-ALL. Consequently, our study revealed the regulatory relationships in the cell cycle related processes of T-ALL and may provide potential therapeutic targets for T-ALL.

## Abbreviations

Cor_sprm: Spearman correlation coefficient
DEG: Differentially expressed gene
DEM: Differentially expressed miRNA
FC: Fold change
FFL: Feed-forward loop
IC50: Half maximal inhibitory concentration
miRNA: microRNA
T-ALL: T-cell acute lymphoblastic leukemia
TF: Transcription factor
